# The central role of climate action in achieving the United Nations' Sustainable Development Goals

**DOI:** 10.1038/s41598-023-47746-w

**Published:** 2023-11-23

**Authors:** Walter Leal Filho, Tony Wall, Amanda Lange Salvia, Maria Alzira Pimenta Dinis, Mark Mifsud

**Affiliations:** 1 UK Consortium on Sustainability Research, Department of Natural Sciences, Manchester Metropolitan University, Chester Street, Manchester, M1 5GD UK; 2 European School of Sustainability Science and Research, Hamburg University of Applied Sciences, Ulmenliet 20, 21033 Hamburg, Germany; 3 Liverpool Business School, Liverpool John Moores University, Liverpool, UK; 4 Graduate Program in Civil and Environment Engineering, University of Passo Fundo, Campus I - BR 285, São José, Passo Fundo, RS 99052-900 Brazil; 5 UFP Energy, Environment and Health Research Unit (FP-ENAS), University Fernando Pessoa (UFP), Praça 9 de Abril 349, 4249-004 Porto, Portugal; 6 Fernando Pessoa Research, Innovation and Development Institute (FP-I3ID), University Fernando Pessoa (UFP), Praça 9 de Abril 349, 4249-004 Porto, Portugal; 7 Centre for Environmental Education & Research, University of Malta, MSD 2080, Msida, Malta

**Keywords:** Climate sciences, Climate change

## Abstract

Sustainable Development Goal (SDG) 13 refers to “Climate Action”. It is one of the 17 goals established by the United Nations in their 2030 Agenda for Sustainable Development. The primary objective of SDG13 is to take urgent action to combat climate change and its impacts. It recognises that climate change is a global challenge that requires immediate attention and concerted efforts from governments, businesses, communities, and individuals worldwide. SDG13 permeates a number of SDGs and also influences them in a significant way. Based on the need to contextualise SDG13 and considering its role as one of the central SDGs, this article outlines the links between SDG13 and the other SDGs. It also reports on a survey involving experts from 61 countries. The findings suggest that even though climate change impacts, particularly extreme weather events, are known to disproportionally affect poorer and minoritized communities, the synergies among related goals and climate justice seem to receive less attention. The article concludes by describing some of the means via which synergies between SDG13 and other SDGs may be achieved.

## Introduction

### SDG13: climate action

Climate change is one of the most pressing global issues of the present time. This has prompted its inclusion among the United Nations Sustainable Development Goals (SDGs), as SDG13, or “Climate Action”. This goal calls for the necessary actions to minimise climate change and address its related impacts. Furthermore, it calls for action to be taken at different organisational levels, with a view to providing a broader response to the problem^1^. As the first volume of the 6th Intergovernmental Panel on Climate Change Assessment Report (AR6) has shown, there is a pressing need to address the drivers of climate change, specifically by a reduction of CO_2_ emissions^2^.

The first target of SDG13 entails strengthening resilience and adaptive capacity to climate related disasters or hazards in all countries^3^. This objective was conceived considering events such as extreme flooding, droughts, heatwaves, wildfires, and other natural disasters in mind, since they affect the livelihood of millions of people worldwide. The aim is to ensure that disaster risk management skills are developed, so that they may assist in the prevention, or at least to reduce the consequences of climate-related events^4^. Another target of SDG13 is to pursue a proper integration of climate change measures and solutions into national and global policies^3^. This may ensure that governments support the related actions or programmes to be implemented regarding climate change which, in turn, may increase a country’s ability to adapt to it^5^. A further target involves improving education and awareness about climate change^6,7^, while increasing capacity building among people and institutions to address it, especially by undertaking concerted adaptation and mitigation action, and interpreting early warning signs^3^. The last target of SDG13 is broken down into 2 subsets. Firstly, it aims to ensure that developed countries are able to mobilize funds of United States Dollar (USD) 100 billion per year to aid developing countries with adaptation and mitigation implementation, and ensuring that the Green Climate Fund remains operational. The second part involves raising the capacity of developing countries and small island nations, while specifically focusing on vulnerable groups such as women, the youth, and marginalized groups of people^3,4^. This is further facilitated by the Global Reporting initiative (GRI) that allows countries to report their contributions and—by doing so—provides a basis for accountability^8^.

### SDG13 and links with the other SDGs

According to the United Nations^9^, tackling climate change in the context of SDG13 will require many urgent actions. The connection of this SDG13 with all other SDGs is reflected in some binding treaties, which include the United Nations Framework Convention on Climate Change (UNFCCC), the Kyoto Protocol, the Doha Amendment and the Paris Agreement, thus illustrating the pre-existing legal obligations towards SDG13^10,11^.

Among its many impacts, climate change is known to lead to reductions in access to drinking water, negatively affecting people’s health (SDG3), limiting their possibilities for income generation (SDG1), also often threatening food security (SDG2)^12^. The 2019 Conference of the Parties of the United Nations Framework Convention on Climate Change—COP 25—in Madrid served as a basis to develop crucial steps in areas such as finance and agriculture^13^, as well as technology, capacity building, the rights of indigenous people, or gender issues^14^, which are some of the many issues which are highly dependent on climatic conditions. COP 26, in Glasgow, showed that Global greenhouse gas (GHG) emissions cuts are still nowhere near where they need to be to keep our climate liveable, and support for the most vulnerable countries affected by climate change is still woefully inadequate. However, COP26 did produce some new "building blocks" to advance implementation of the Paris Agreement, which could help put the world on a more sustainable, low-carbon pathway^15^. COP 27, in the Egyptian coastal city of Sharm el-Sheikh, closed with a ground-breaking agreement to provide money to help countries that have been severely affected by climate-related disasters, such as floods, droughts, and others^16^. This was widely praised as a historic decision. All these steps are to be paralleled by actions intended to further reduce GHG emissions from human activities, reducing the impacts of climate change in building climate resilience. The United Nations Department of Economic and Social Affairs stated that even though the lockdowns associated with the COVID-19 pandemic have led to a 6% drop in GHG emissions in 2020, this is not enough to achieve the annual reductions necessary to limit global warming to 1.5º C^17^. Financing of climate action has increased, but continues to be exceeded by investments in fossil fuels^17^. Also, the progress in meeting disaster risk reduction targets is rather slow between countries, which suggests that more cooperation (SDG17) is needed^18,19^.

Air pollution (SDG3), water scarcity (SDG6), food security (SDG2), land use (SDG15) and sustainable energy (SDG7) are important SDGs which are to a considerable extent associated with climate change. Therefore, climate issues need to be carefully considered in their implementation. Liu et al.^20^ highlight that the advantages of joint efforts to implement climate policies need to be recognized by policy-makers, since all interactions are influenced by socioeconomic factors.

A recent review of the implementation of the SDGs and of the interrelations among the goals undertaken by the United Nations^9^ revealed that many SDGs integrate economic growth, environmental protection and social well-being dimensions, applying both to high-income as to low-income countries. Therefore, further attention to climate change is necessary to achieve fairer economic and social prosperity^21^. It is also about considering that co-benefits, i.e., synergies resulting from climate change mitigation actions delivering non-climate benefits, are important in climate policies, since they are able to lead to improvements in areas such as energy, or in forests’ protection. However, trade-offs, i.e., higher energy prices or more people risking hunger, represent risks and adverse side-effects that must also be accounted for in designing actions^20,22^. Cohen et al.^22^ report the importance of considering both co-benefits and trade-offs involved, i.e., co-impacts, in climate actions, aiming to maximise the former and minimize the latter. There is also a need to search for synergistic outcomes meeting multiple objectives. Each of the 17 SDGs have synergies with climate change, while a few have no direct trade-offs, i.e., SDGs 3, 4, 5, 12 and 13^18^.

**Figure 1:**
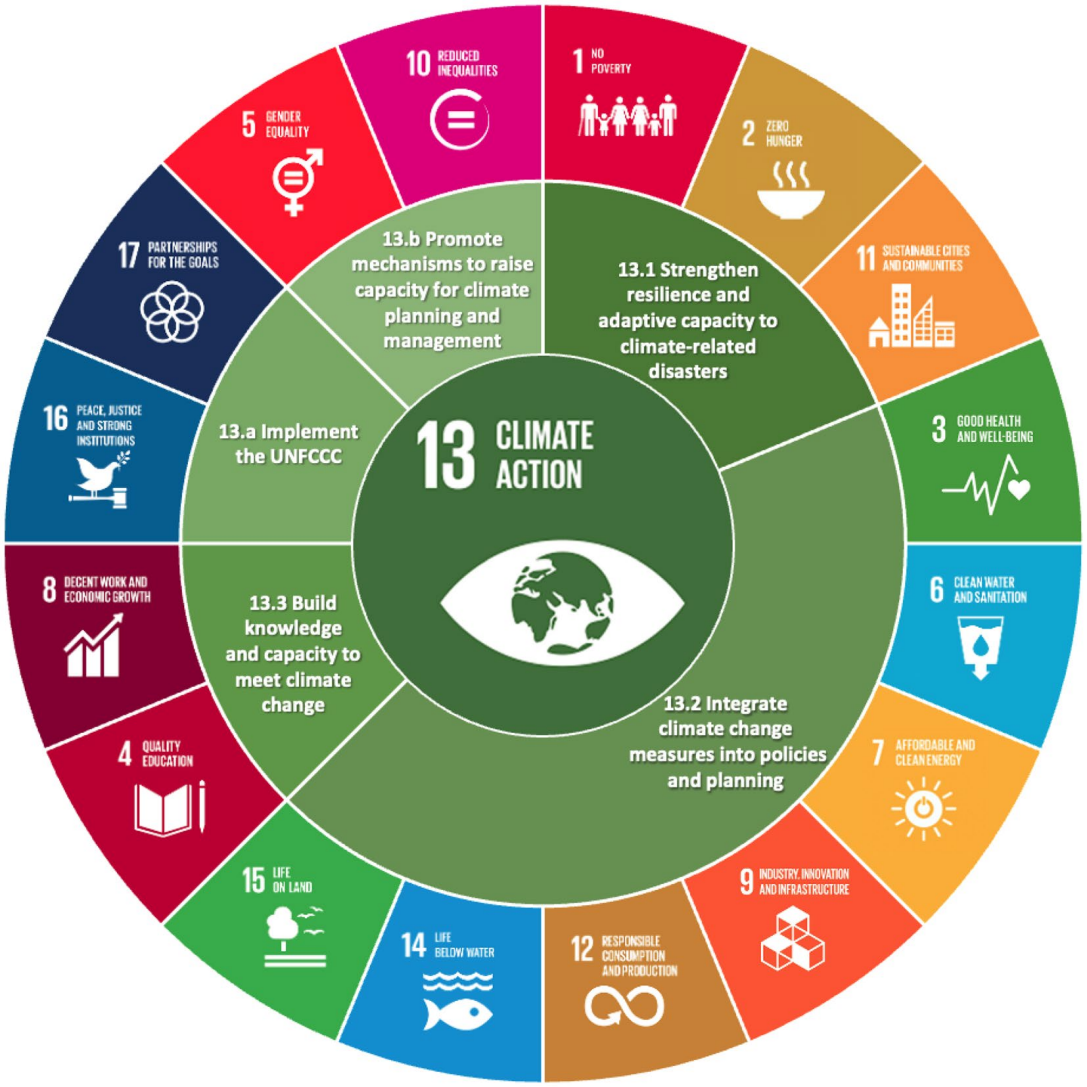
Relation among targets of Climate Action and the SDGs. Source: Authors.

Based on this context and on the need to further discuss the relations among climate change and all SDGs, Fig. 1 explores an approach that relates each target of the goal on Climate Action (SDG13) to the other SDGs. Disaster risk reduction strategies and its implications (target 13.1), for instance, are covered directly by some of the targets of SDGs 1, 2 and 11, in terms of building resilience of the poor and those in vulnerable situations, implementing resilient agriculture, and reducing the number of affected people, while promoting more policies and plans towards mitigation and adaptation to climate change.

Target 13.2 covers the integration of climate change measures into policies and planning and can be related to a wide range of topics—and consequently, can be relevant to all SDGs. Yet, not all of SDGs have clear references to climate change or climate action efforts, but all would benefit from such policies and plans.

The ability to adapt to climate change and promote resilience encompasses the impacts on health (SDG3) and on provision of water services (SDG6). SDGs 7, 9 and 12 also have important roles in fostering more sustainable strategies to contribute to reduced greenhouse gas emissions—from renewable energy and energy efficiency to innovative approaches in production, consumption, and industrial services. The impacts of climate change on aquatic and terrestrial ecosystems are numerous, including desertification, ocean acidification, and the consequences to biodiversity. Therefore, SDGs 14 and 15 are also expected to benefit from a greater consideration to climate change in local and national plans and strategies.

The process of building knowledge and capacity to meet climate change is explored in target 13.3 which outlines the advantages of a greater integration of climate issues in the educational curricula and in capacity-building programmes. SDGs 4 and 8 are also closely associated with these aspects, having one or more targets that demand the acquisition of knowledge and skills to promote sustainable development or resource efficiency (e.g., targets 4.7 and 8.4), in addition to the importance of having the workforce prepared to apply climate action measures in various sectors.

The targets related to the means of implementation (13.a and 13.b) rely on strong institutions and international partnerships (SDGs 16 and 17) for a mobilization of the resources associated with the UNFCCC, and highlight the importance of mechanisms to support climate change planning and management, hence contributing to SDGs 5 and 10^19,23^.

## Methods

To further the understanding of the influences of SDG13 on the other SDGs, an international exploratory study was carried out. The European School of Sustainability Science and Research (ESSSR) and the Inter-University Sustainable Development Research Programme (IUSDRP) disseminated a survey among their networks, asking expert respondents to answer three questions: (i) their country (closed question, list of countries as options were provided); (ii) their primary position (closed question, with options Research, Teaching, Administration and Other); and (iii) “To which extent does the implementation of SDG13 (Climate Action) positively influence the implementation of the other SDGs?”. A Likert-type scale (no influence, a little, moderately, to some extent, to a great extent) was presented as response option for all goals (SDGs 1–17 listed, except for SDG13).

An invitation to contribute to the survey was widely shared among the networks of the ESSSR and IUSDRP, i.e., meaning researchers and teaching and management staff and other representatives of universities across all geographical areas. They were also encouraged to share the invitation across their institutional networks, helping to disseminate it further. Since participation in the survey was voluntary, the authors had no influence on the willingness of participants to take part on it. The only criteria specifically mentioned in the survey invitation was for respondents to be familiar with the study topic, i.e., Climate Action and the other SDGs.

The internet-based survey, the method chosen for this study, is a standard procedure which requires no specific ethics consent in Germany, as confirmed by the German Association of Medical Ethics Committees. The survey implementation followed the procedures and guidelines used in surveys in Germany and at HAW Hamburg, the lead organisation. Informed consent was sought from all participants, who voluntarily agreed to proceed with the completion of the questionnaire, anonymised so that no personal details were stored.

### Ethics statement

The nature of the research, the methods used, and the fact that no personal data was stored or can be traced back to individuals, conforming with GDPR standards, means that the study is not subject to an ethics permit as specified by the Association of Medical Ethics Committee in Germany, the body responsible for such assessments in the country which led the study.

## Results and discussion

**Figure 2:**
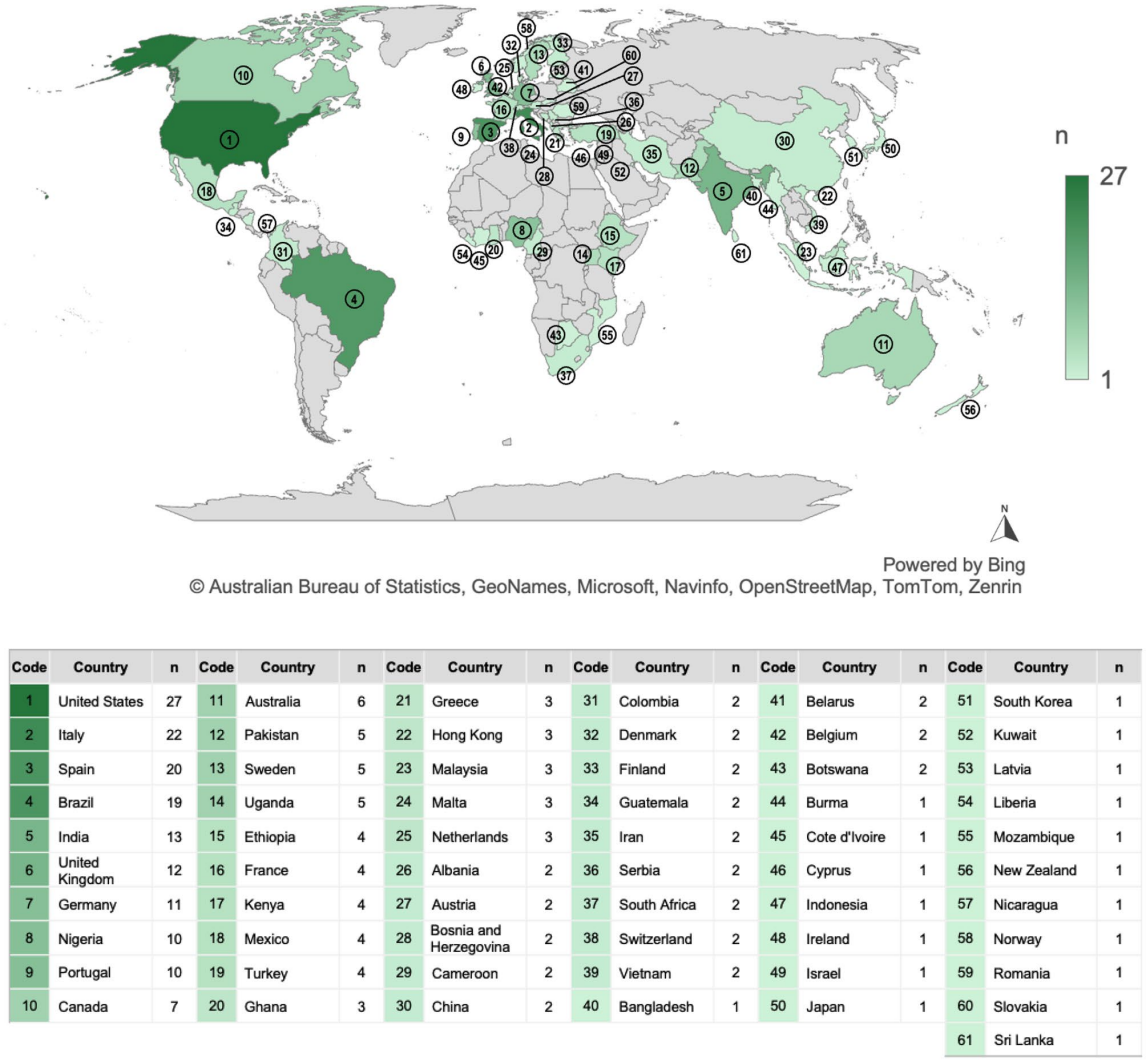
Distribution of responses around the world.

The survey received 260 responses from respondents in all regions, as shown in Fig. 2. The sample cannot be regarded as representative but allows a rough profile of current trends to be built on an international scale. In total, there were 61 countries participating in this study, whose responses were collected between September–October 2021. As primary position in the higher education sector, around 47% of the respondents are researchers, followed by 33% engaged in teaching activities and 9.2% working on administrative functions. The remaining percentage refers to colleagues working on combined positions (e.g., teaching and research) as well as management, consulting and other climate-related roles.

**Figure 3:**
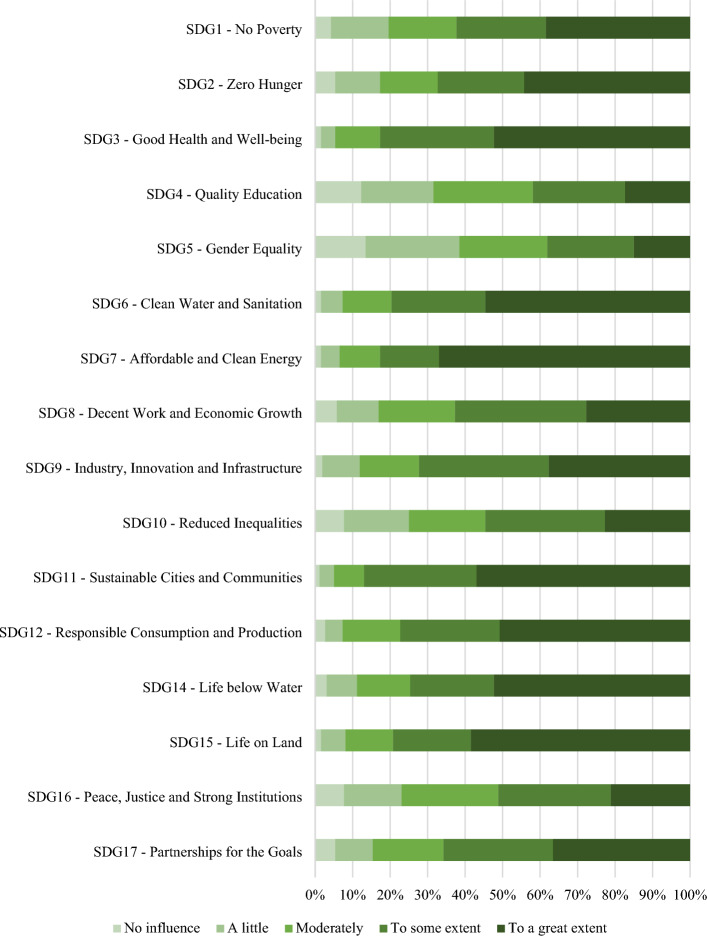
Extent the implementation of SDG13 influences positively the implementation of the other SDGs.

Figure 3 summarises the results of this investigation. Generally, the respondents acknowledged the important influence SDG13 has on all other SDGs. For a set of goals, the influence seems to be more pronounced: SDGs 3 (Good health and well-being), 6 (Clean water and sanitation), 7 (Affordable and clean energy), 11 (Sustainable cities and communities), 12 (Responsible consumption and production), 14 (Life below water) and 15 (Life on land) received over half of the responses in the highest category of influence. SDG7 stands out with the higher percentage in the group (67%). Sustainable energy and renewable energy investments are extensively discussed along with climate change matters^24,25^, and the synergies both goals have is clear in all reports on the topic. In addition to health, which has been receiving increased attention in terms of the impacts associated with climate change^26^, these group combines most goals directly associated with the Planet dimension of the 2030 Agenda.

For other SDGs—especially those associated with social and economic aspects, the perception of influence is slightly less pronounced, with responses distributed among the two highest response categories: SDGs 1 (No Poverty), 2 (Zero Hunger), 8 (Decent work and economic growth), 9 (Industry, innovation and infrastructure), 17 (Partnerships for the goals).

The final group is formed by SDGs 4 (Quality education), 5 (Gender equality), 10 (Reduced inequalities) and 16 (Peace, justice and strong institutions). These were the only goals to receive over 20% of responses in the two lower categories of influence. In Fig. 3, Quality education and Gender equality stand out for having the highest percentages of responses indicating “no influence” of SDG13, and the lowest percentages of responses supporting a great influence of the climate goal. Even though climate change impacts, particularly extreme weather events, are known to disproportionally affect poorer and minoritized communities, the synergies among related goals and climate justice seem to receive less attention^18,27^.

## Conclusions: the way ahead

As this communication has shown, SDG13 on climate action is a central SDG that seeks to strengthen resilience and reduce vulnerability to climate-related hazards and natural disasters. Climate action is not only about creating a low-carbon, climate-resilient world by reducing GHG emissions, investing in renewable energy, and improving climate adaptation measures. It also involves empowering individuals, communities, and nations to make informed decisions and develop the necessary skills to manage climate risks. To achieve SDG13, governments, businesses, and civil society must work together to develop and implement ambitious climate policies, which may improve the lives of the populations, apart from emissions reductions.

As this article has shown, there are many direct and indirect interrelations between SDG13 and other SDGs. However, these are not well understood. As a result, many opportunities for potential synergies to integrate climate action with other SDGs are being missed. But this gap may also represent an opportunity, since it can be bridged. Some of the measures which may be deployed in order to address this problem, may include:

(i) Greater consideration to climate change and climatic aspects in plans aimed at increasing sources of local income (SDG1) and food production (SDG2). For instance, plans to diversify subsistence agriculture in Africa need to consider the use of drought resistant crops, taking into account increasing temperatures and longer dry periods.
(ii) A due emphasis on climate issues in the design of, and adjustments to, public health plans and policies, so as not only to pay due attention to the many vector-borne diseases associated with climate change, but to also foster the health of populations affected by extreme events which are known to be associated with high morbidity (SDG3).
(iii) A wider awareness of the fact that habitat protection and life quality (SDG14, SDG15) in cities (SDG 11) are associated with climatic conditions, which in some cases, may also be associated with conflict (SDG16). This should trigger a greater motivation to consider an emphasis to climate change, which may support their delivery.
(iv) New forms of governance where climate change plays a more central role, and not a marginal one, as it is largely the case at present.

The following recommendations may be followed by policy-makers:

- Increase funding for climate and sustainability research, including the social sciences’ role in understanding and influencing behaviour change.
- Foster partnerships between universities, government agencies, and industry to promote the development of sustainable technologies and climate solutions.
- Support programmes that encourage student and faculty involvement in climate action projects, such as sustainability committees or climate action groups.
- Mandate the creation of a climate action plan for each university, setting clear targets for reducing greenhouse gas emissions and establishing monitoring and reporting mechanisms.
- Establish systems for monitoring and evaluating the implementation of sustainability and climate action initiatives at universities.
- Use the data from this article to refine policies, allocate resources effectively, and scale successful initiatives.

Further measures could be used, but by taking these steps, policy-makers can effectively help universities to be leaders in the transition to a more sustainable and climate-resilient society.

Finally, it is important that a greater coordination of the work of the various United Nations agencies and national organisations responsible for the implementation of the SDGs is pursued, so as to maximise the outcomes of financial investments already made and yield the expected benefits.

## Data Availability

The datasets generated during the current study are available from the corresponding author on reasonable request.
